# Gas Mixing and Final Mixture Composition Control in Simple Geometry Micro-mixers via DSMC Analysis

**DOI:** 10.3390/mi10030178

**Published:** 2019-03-07

**Authors:** Stavros Meskos, Stefan Stefanov, Dimitris Valougeorgis

**Affiliations:** 1 Institute of Mechanics, Bulgarian Academy of Sciences, Acad. G. Bontchev St. bl. 4, 1113 Sofia, Bulgaria; stefanov@imbm.bas.bg; 2 Department of Mechanical Engineering, University of Thessaly—Pedion Areos, 38334 Volos, Greece; diva@mie.uth.gr

**Keywords:** binary gas mixing, micro-mixer, DSMC, splitter, mixing length, control mixture composition

## Abstract

The mixing process of two pressure driven steady-state rarefied gas streams flowing between two parallel plates was investigated via DSMC (Direct Simulation Monte Carlo) for different combinations of gases. The distance from the inlet, where the associated relative density difference of each species is minimized and the associated mixture homogeneity is optimized, is the so-called mixing length. In general, gas mixing progressed very rapidly. The type of gas surface interaction was clearly the most important parameter affecting gas mixing. As the reflection became more specular, the mixing length significantly increased. The mixing lengths of the HS (hard sphere) and VHS (variable hard sphere) collision models were higher than those of the VSS (variable soft sphere) model, while the corresponding relative density differences were negligible. In addition, the molecular mass ratio of the two components had a minor effect on the mixing length and a more important effect on the relative density difference. The mixture became less homogenous as the molecular mass ratio reduced. Finally, varying the channel length and/or the wall temperature had a minor effect. Furthermore, it was proposed to control the output mixture composition by adding in the mixing zone, the so-called splitter, separating the downstream flow into two outlet mainstreams. Based on intensive simulation data with the splitter, simple approximate expressions were derived, capable of providing, once the desired outlet mixture composition was specified, the correct position of the splitter, without performing time consuming simulations. The mixing analysis performed and the proposed approach for controlling gas mixing may support corresponding experimental work, as well as the design of gas micro-mixers.

## 1. Introduction

Micro-scale gas mixing is of major theoretical and industrial interest in the development and optimal design of gaseous MEMS/NEMS (micro/nano-electro-mechanical systems) devices [1]. Micro-mixers can be considered as the miniaturized counterparts of macro-mixers. When chemical reactions take place, they are referred to as micro-reactors. Most commonly, they are encountered as part of a larger assembly of micro-channels and micro-devices in various applications such as micro-pumps, micro-turbines, micro-engines and micro-sensors. The detailed investigation of the effects of the geometry, boundary conditions and operating conditions on mixing is essential in the design of these devices.

The computational analysis of gaseous flows in MEMS devices operating under different conditions in non-equilibrium flow regimes cannot be based on classical continuum models of fluid motion because the continuum assumption that the flow is locally in the near-equilibrium state is no longer valid. Thus, numerical methods that solve the Boltzmann equation such as the Discrete Velocity and the Lattice Boltzmann methods, as well as particle-based methods such as the Molecular Dynamics and the Direct Simulation Monte Carlo (DSMC) methods are used instead. The DSMC method [2,3] is arguably the most common and is employed in this work.

Most of the available work has focused on the investigation of the flow characteristics of already fully mixed rarefied gases inside micro-channels of various cross-sections. The flow of monatomic binary gas mixtures in the whole range of the Knudsen number, through long capillaries of circular and rectangular cross sections has been studied in [4,5] and [6,7] respectively. A comparative study between computational and experimental results for flows in long microchannels has been presented in [8]. All the aforementioned works solve the Boltzmann kinetic equation, for fully developed flows, replacing the collision term with the McCormack model for gas mixtures [9]. Time-dependent flow and heat configurations of binary gas mixtures have also been investigated based on the DSMC method [10,11,12,13]. The flow rates of transient binary gas mixture flows through circular capillaries of finite length, including some comparison with corresponding experimental results are presented in [10,11]. The associated separation effects for flows though short and long capillaries were analyzed in [12] and [5] respectively. The transient heat flow response of a binary gaseous mixture confined between coaxial cylinders for various values of temperature difference was studied in [13].

Contrary to binary gas flows of premixed gases, the process of gas mixing of two gases entering the mixing chamber from different inlets has attracted less attention. One of the first studies performed was in [14], where the dependence of the mixing length on the pressure ratio between the inlets and the outlets, as well as on the inlet velocities was investigated. The tested configuration consisted of two parallel gas streams of H_2_ and O_2_ entering the mixing chamber, with the outlet pressure constantly kept at 50 kPa. It was found that the mixing length was increased by increasing the pressure ratio between inlets and outlets, and between the two inlets. The same flow setup has also been examined in [15] testing the mixing of CO and N_2_ while the outlet pressure was kept at zero (no back-flow). In this work, the terms, relative density difference and mixing coefficient were introduced in order to better describe the mixing process. It has been found that the mixing length is inversely proportional to the gas temperature and the Knudsen number, while it is proportional to the Mach number. It has been also shown that the wall characteristics have little effect on the mixing length. The mixing of CO and N_2_ in a T-shaped micro-mixer by keeping the outlet pressure low enough to prevent back-flow is presented in [16]. It was found that at a higher Knudsen number the mixing length is reduced, while increasing the inlet pressure resulted in an increased mixing length. Also, increasing the flow and the wall temperatures resulted in reduced mixing lengths, with the wall temperature effect being the more significant one. In [17] an effort was made to improve the mixing efficiency by attaching two bumps at the upper and lower walls, just after the end of the inlet plate separating the two gas streams. In [18] the mixing process of N_2_ and CO in a microchannel was investigated and the results showed that the mixing length increased when the inlet-outlet pressure difference was increased, while it reduced when the pressure ratio of the two species at the inlet was increased. More recently, CO and N_2_ mixing at an angle by applying Y-shaped inlets was investigated [19]. In the same work, the effect of replacing a “larger” micro-mixer with many “smaller” ones was also studied. Finally, the influence of gas rarefaction on the diffusive mass transfer in slip and transitional regimes has been examined [20]. All the works related to gas mixing employ the DSMC method.

The present work focused on gas mixing. More specifically, the mixing of two parallel gas streams entering a micro-channel was simulated using the DSMC method. The influence of the wall accommodation coefficients, the temperature, the molecular mass and diameter ratios of the two species and the implemented intermolecular collision model on the mixing process was investigated. Various gas mixtures were simulated and in all cases the mixing length and the flow field in the mixing micro-channel were computed. Then, another plate parallel to the micro-channel walls was positioned downstream at a lesser distance than the mixing length in order to split the partially mixed stream into two outlet streams with specified compositions. In this way it was possible to control the output mixture composition by properly positioning the splitter while keeping the inlet conditions fixed. The relationship between the splitter position and the outlet mixture composition was investigated. The micro-mixer geometry with the associated flow setup is given in Section 2, followed by a brief description of the DSMC scheme and the numerical parameters involved in Section 3. Then, the detailed mixing length analysis is presented in Section 4, while the proposed methodology for controlling the output mixture composition is given in Section 5. The manuscript concludes with some final remarks in Section 6.

## 2. Micro-Mixer Geometry and Flow Setup

**Figure 1:**
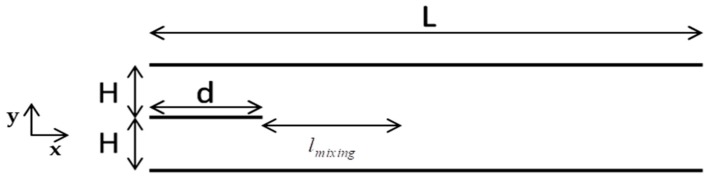
Schematic of gas micro-mixer.

Consider the pressure driven two-dimensional gas mixing flow of two rarefied gases into a rather simple micro-mixer, as shown in Figure 1. It consisted of two parallel plates of finite length L, located at y=0 and y=2H, and of a third parallel plate of length d<L/3, located at y=H. The origin of all three plates was at x=0. The light gas, denoted by the subscript “1” entered from above (H≤y≤2H), while the heavy gas, denoted by the subscript “2” entered from below (0≤y≤H). Furthermore, the inlet pressures $P_{\mathrm{in}}^{1}$ and $P_{\mathrm{in}}^{2}$ at x=0 were fixed, while the exit pressure at x=L was set equal to zero (expansion into vacuum). The inlet and wall temperatures are $T_{0}$ and $T_{w}$ respectively, with the latter one being the same for all plates. Maxwell diffuse-specular boundary conditions were considered at the walls. To cover a wide range of mixing with regard to the molecular mass of the involved gases the following combinations were considered: CO-N_2_, Ne-Ar, He-Ne, He-Ar, and He-Xe. The molecular mass ratio of the light over the heavy species ranged from 1 for CO- N_2_ down to 0.03 for He-Xe.

Due to the imposed pressure difference there was a steady-state flow of the two species along the channel, while in parallel, due to molecular diffusion, the two species were mixed. The areas x≤d and x>d were considered as the inlet and mixing zones respectively and the corresponding chambers were defined as the inlet and mixing chambers. The two species start mixing, upon entering the mixing chamber. The mixing process progressed gradually, mainly in the flow direction x up to some distance, denoted by $L_{\mathrm{mix}}$, where the two species were considered as mixed, provided that some mixing criteria related to the homogeneity of the mixture was fulfilled. The length $l_{\mathrm{mix}}=L_{\mathrm{mix}}-d$ defined as the mixing length, characterized each mixing process and is of major importance in the present work.

**Figure 2:**
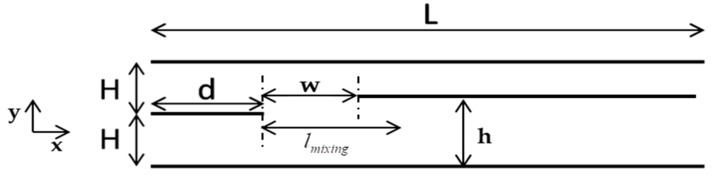
Schematic of gas micro-mixer with splitter.

Next, in order to be able to control the composition of the binary mixture produced, in terms of its two species, another plate, the so-called splitter, was added parallel to all other plates, with its origin at some point (x,y)=(d+w,h), where $w<l_{\mathrm{mix}}$ and 0<h<2H, as shown in Figure 2. All other flow conditions remained the same. Positioning the splitter within the specified zone of the flow domain, where the mixing process was still in progress but not yet completed, resulted in having two outlet mixture streams of specific compositions, different from the fully mixed one without the splitter. Therefore, by locating the splitter at different points inside the mixing zone, and by fixing w and h accordingly, the mixing process could be controlled and the desired mixture composition at the outlet could be recovered. The areas x≤d, d<x≤d+w and x>d+w were considered as the inlet, premixing and mixing zones respectively.

As noted above, a mixture is considered as fully mixed, provided that some mixing condition, which is directly connected to the number densities of the mixture components, is fulfilled. The orthogonal flow domain 0≤x≤L, 0≤y≤2H is discretized into i=1,2,…,I rows and j=1,2,…,J columns, while the number density of each species at the (i,j) cell is denoted as $n_{i,j}^{a}$, with a=1,2. Then, the relative number density difference is defined as:

(1) $$\xi_{j}^{a}=\frac{1}{I}\sum_{i=1}^{I}\frac{\left|n_{i,j}^{a}-{\bar{n}}_{j}^{a}\right|}{{\bar{n}}_{j}^{a}}$$

where ${\bar{n}}_{j}^{a}$ is the average number density of species a=1,2 of all cells in column j, given by:

(2) $${\bar{n}}_{j}^{a}=\frac{1}{I}\sum_{i=1}^{I}n_{i,j}^{a}$$

In this work the relative number density difference $\xi_{j}^{a}$, will be referred to from now on as “$RDD^{a}$”. As the species number density at each cell of a channel column tends to coincide with the species average number density of the column, the $RDD^{a}$ tends to zero. It is evident that the relative density difference is a measurement of the homogeneity of the mixture. Ideally, perfect mixing implies that the relative density difference of both components is equal to zero. Since this is not practically achievable, the mixtures were considered fully mixed provided that $RDD^{a}<\varepsilon$, where, depending upon the flow setup, ε took values from 0.005 up to 0.025 or on a percentage basis from 0.5% up to 0.25%. The mixing length $l_{\mathrm{mix}}^{a}$ of each mixture component a=1,2, is the distance, where the mixing criteria is fulfilled, while if the fully mixed criterion, for several reasons, cannot be satisfied, the mixing length $l_{\mathrm{mix}}^{a}$ is the distance, where the corresponding minimum values of the $RDD^{a}$ are observed. In general, $l_{\mathrm{mix}}^{1}\neq l_{\mathrm{mix}}^{2}$ and therefore, the mixing length of the mixture is specified as $l_{\mathrm{mix}}=\max\left(l_{\mathrm{mix}}^{a}\right)$. Similarly, the mixture number density difference is defined as $RDD=\max\left(RDD^{a}\right)$. It is noted that the definition of the RDD according to Equations (1) and (2) is an extension of the one presented by Wang and Li [15], with the present definition providing, as discussed in Appendix A, more reliable results free of computational noise.

An associated quantity of interest in the present work is the cell molar fraction of the mixture defined as:

(3) $$C_{i,j}^{a}=n_{i,j}^{a}/n_{i,j}$$

where $n_{i,j}=n_{i,j}^{1}+n_{i,j}^{2}$ is the cell total number density and obviously,$C_{i,j}^{1}+C_{i,j}^{2}=1$. The average column molar fraction ${\bar{C}}_{i,j}^{a}$ is defined similar to the corresponding number density in Equation (2).

Following the description of the numerical scheme in Section 3, the flow configurations of Figure 1 and Figure 2 were simulated and results are presented in Section 4 and Section 5, respectively. The results in Section 4 relate to the computation of the mixing length in terms of all flow parameters, while the results in Section 5 relate to the control of the mixing process and to the production of gas mixtures of specific compositions. 

## 3. Computational Scheme

The DSMC method, which is used in the present study, is a well-known particle-based stochastic method that simulates gas flows very efficiently across a wide range of the Knudsen number. The main principle of the method is decoupling the collisions and the motion of particles. This is achieved by considering a time step smaller than the amount of time a particle travels a mean free path with the most probable velocity. Each simulated particle represents a huge number of real molecules, usually greater than 10^18^. The computational domain is divided into cells. Particles are moving through the cells, collisions are occurring only between particles within the same cell and finally, molecular speeds and number densities are sampled at each cell. This process is repeated at each time step and the simulation continues in time until steady-state conditions are reached. There are two possibilities for averaging. The first is done by time averaging a large number of time steps after reaching the steady-state. This averaging method is sufficient when only the steady-state solution is of interest. However, in order to derive accurate results for the transient period, an ensemble averaging method is required as well, where many independent simulations are performed and results are additionally averaged at the same time steps by ensemble averaging over all simulations. An in-house DSMC code was developed and employed in this work, which combines both time and ensemble averaging methods. For a detailed description of the method, the reader is referred to [21,22,23].

In Table 1, the computational properties of the implemented DSMC method, applied in all simulations unless otherwise stated, are presented. The grid consists of square cells with Δx=Δy=1/30. For the sampling procedure, a coarser grid was used which was exactly half of the original one. For the collision procedure, the fine grid was used. The time step was always less than one-third of the mean free time and, in particular, was set approximately equal to the time needed for a particle to travel the shortest length of a cell with the most probable velocity. For example, for the mixing flow case of CO-N_2_, it was set at 0.2 ns. The weight factor is defined as the ratio of real number density to the simulated number of particles and was set in a way that would result in fluxes approximately equal to 200 particles per cell per time step. Note that this number represents only the calculated inlet flux and not the net flux.

The pressure and temperature at the inlets (x=0) were kept constant. The number density is derived from the equation of state and then, the fluxes are calculated as [3]:

(4) $$\dot{N}=\left[n\exp(-s_{n}^{2})\pm \sqrt{\pi }s_{n}\left\{1\pm erf(s_{n})\right\}\right]/\left(2\sqrt{\pi }\beta \right)$$

where n is the weighted number density, $s_{n}=u_{0}\beta$ is the dimensionless inlet velocity, with $u_{0}$ being the inlet dimensional velocity and $\beta =\sqrt{m/\left(2k_{B}T_{0}\right)}$ is the inverse of the most probable speed ($k_{B}$ is the Boltzmann constant). In this study, the inlet bulk velocity is zero and Equation (3) is reduced to:

(5) $$\dot{N}=n/\left(2\sqrt{\pi }\beta \right)$$

Initially, vacuum conditions are imposed inside the computational domain and the initial molecular velocities are sampled from the Maxwellian distribution.

## 4. Mixing Length Analysis

The mixing length in terms of various parameters affecting the mixing process was computed for the flow configuration shown in Figure 1. More specifically, the parameters involved included the effect of the channel length, the accommodation coefficient, the wall temperature, the intermolecular collision model and the molecular mass ratio of the mixture components. In the base case scenario, the half distance between the plates is H=1 μm, the channel length L=8 μm and the length of the inlet middle plate d=2 μm. The working gases are CO and N_2_ having a molecular mass ratio equal to one. Also, the inlet pressure and temperature are $P_{\mathrm{in}}^{1}=P_{\mathrm{in}}^{2}=P_{\mathrm{in}}=0.2$ atm and $T_{0}=300$ K, while the temperature of all plates is taken as equal to the inlet temperature ($T_{w}=T_{0}$). Finally, purely diffuse reflection is considered at the walls (the accommodation coefficient is equal to one) and the intermolecular collision model is the variable soft sphere (VSS) model. In the base case scenario, the reference Knudsen number is approximately 0.3 and the flow is in the transition regime.

**Figure 3:**
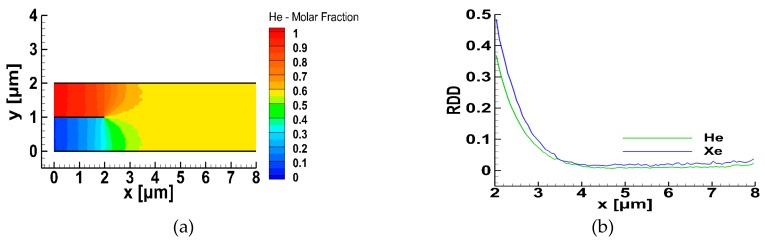
Gas mixing of He and Xe in the micro-mixer: (**a**) molar fraction contours of He, and (**b**) evolution of the relative density difference of He and Xe in the mixing zone.

To obtain an initial view of the gas mixing in the micro-mixer, the flow of He and Xe, keeping all other parameters as in the base case scenario, was considered. The corresponding contours of the molar fraction of He and the variation of the relative density difference of the two species ($RDD^{a}$), are plotted in Figure 3a,b respectively. At x=0, the molar fraction of He is very close to one in the upper inlet and close to zero in the lower one. Then, the two species start to mix, even in the inlet zone, where the molar fraction of He is gradually decreased in the upper part, and increased in the lower part reaching, at the end of the inlet zone (x=2 μm), values of approximately 0.7 and 0.3, respectively. On entering the mixing zone, gas mixing is rapid and in a distance of about x=4 μm the two species were fully mixed. The corresponding variation of the relative density difference of He and Xe in the mixing zone is also shown. Both $RDD^{a}$ rapidly decreased reaching a minimum value at approximately x=4.2 μm, and then they slightly increased up to the channel end. The molar fraction of He in the resulting fully mixed He-Xe mixture was approximately 0.57.

**Figure 4:**
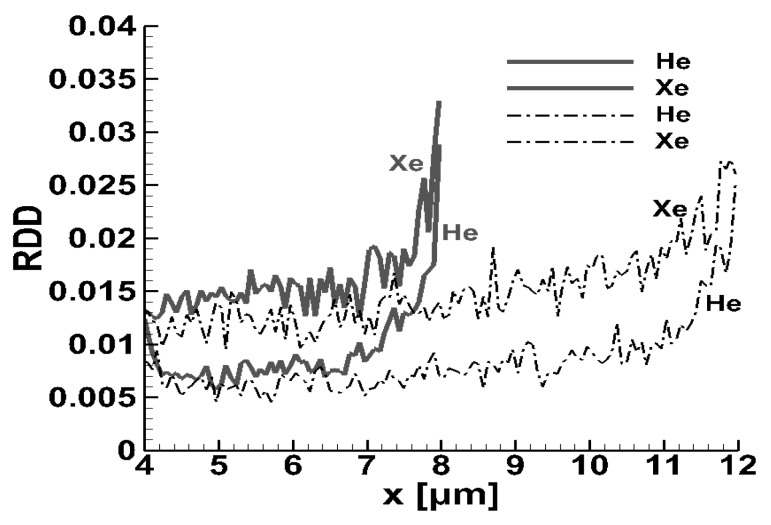
Detailed view of the evolution of the relative density difference of He and Xe in the downstream part of the mixing zone of micro-mixers with a total length of 8 μm (solid grey) and 12 μm (dash-dot black).

A more detailed view of the behavior of the species’ relative density difference in the downstream part of the mixing chamber is shown in Figure 4, where the $RDD^{a}$ are plotted for the flow of He and Xe through micro-mixers with L=8 μm and L=12 μm, keeping all other parameters according to the base scenario. In both cases, the relative density differences rapidly reduced as the gases entered the mixing chamber (x>2), reaching some minimum values at 4<x<5. The distances where the minimum $RDD^{a}$ are observed correspond to the mixing lengths $l_{\mathrm{mix}}^{a}$. Then, as x is further increased, the relative density differences slightly increase up to the end part of the channels, where they rapidly increase, due to gas separation plus end-effects, which strongly affect the homogeneity of the mixture. The $RDD^{a}$ curves of He and Xe, for both channel lengths, never cross each other. Overall, it is noted that for both channel lengths, L=8 μm and L=12 μm, the evolution of the relative density difference was similar and more importantly, the minimum values of $RDD^{a}$ and the corresponding mixing lengths $l_{\mathrm{mix}}^{a}$ were identical. This observation clarifies that the mixing process is independent of the channel length, provided that the channel is longer than some critical value, allowing the evolution of the mixing process. It is also clarified that taking L=8 μm in the base case scenario is adequate in order to investigate the effect of all other parameters.

**Figure 5:**
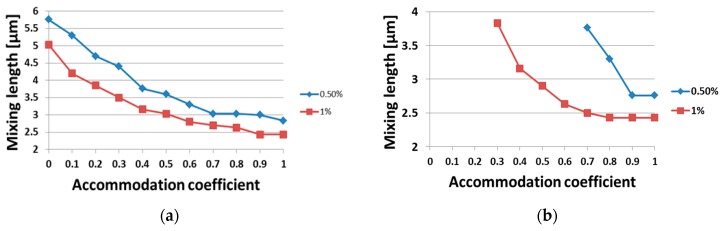
Mixing length variation over different accommodation coefficients α at walls: (**a**) all walls have the same α∈[0,1] and (**b**) the upper wall has α∈[0,1], while all other walls have α=1.

Next, the effect of the gas–surface interaction on the mixing process was examined for the base case flow scenario. In Figure 5 the mixing length $l_{\mathrm{mix}}$ in terms of the accommodation coefficient α∈[0,1] is plotted. The limiting cases of α=0 and α=1 correspond to purely specular and diffuse reflection, respectively. In Figure 5a, the same accommodation coefficient is applied to all micro-mixer walls, while in Figure 5b the accommodation varies only in the upper wall (y=2H) and is kept equal to one in all other walls. Also, it is assumed that both gases have the same α. Results are presented for two values of the mixture relative density difference, RDD, namely 0.5% and 1%. It is clearly seen that α has a significant effect and more specifically, the mixing length increases as the reflection becomes more specular. For RDD<1% the mixing length approximately doubled when the accommodation coefficient was reduced from α=1 to α=0. As expected, the mixing length also increased as the homogeneity criterion was reduced from 1% to 0.5%. This reduction increased the mixing length about 1 μm in Figure 5a and even more in Figure 5b. Also, in Figure 5b, the required threshold values of RDD<1% and RDD<0.5% were not recovered at all for α<0.3 and α<0.7, respectively and therefore are not shown in the figure. It may be clearly stated that in the transition regime the gas–surface interaction plays a significant role in the mixing process, which may be more important than the interaction between particles.

Next, the effect of the temperature of the walls was investigated. In Table 2, the mixing length $l_{\mathrm{mix}}$ of the mixture is tabulated with the temperature of the walls being uniform and equal to $T_{w}=300$, 450 and 600 K. The inlet temperature is always kept at $T_{0}=300$ K. In addition to the mixing length, the corresponding $C^{1}$ and $RDD^{a}$ values are provided. The largest $RDD^{a}$ is also the RDD of the mixture. In the last column of Table 2, a mean value of the relative density difference, denoted by 〈RDD〉, is also given. It has been computed by averaging all $RDD^{a}$ in the mixing chamber from x=4.5 μm up to x=7 μm, i.e., approximately from the fully mixed distance up to the region just before the channel end. This mean value provides an estimate of the overall mixture homogeneity downstream of the mixing length. It is seen that a significant increase in the walls temperature yields a rather small decrease of the mixing length and the molar fraction of CO remains almost constant at 50%. The corresponding minimum RDD of the mixture increased slightly from 0.59% at 300 K to 0.75% at 450 K and 600 K, indicating that the mixture became less homogenous. This is further strengthened by the mean value of the relative density difference 〈RDD〉, which increased from 0.76% to 1.0%. Similar conclusions were drawn based on the corresponding results for flow mixing of He-Xe presented in Table 3. Overall, it may be stated that the temperature of the walls had a minor effect on the mixing process. 

Computations were performed with several intermolecular collision models and their effect on the gas mixing results is reported next. In Table 4, the mixing length of the mixture is tabulated employing the hard sphere (HS) and the variable hard sphere (VHS) models, proposed by Bird [24], as well as the variable soft sphere (VSS) molecular model, proposed by Koura and Matsumoto [25]. The corresponding $C^{1}$ and $RDD^{a}$ values are also provided. The temperature of the walls was kept equal to the inlet temperature at 300 K. It is interesting to see that the mixing length varied significantly between the models. Compared to the VSS model, which is the model in the base case scenario, the mixing lengths with the VHS and HS models increased 12.4% and 17.4%, respectively, with the RDD remaining the same at approximately 0.60%. This is a significant increase in the mixing length considering that all wall and inlet/outlet boundaries were at isothermal conditions.

In order to further investigate the effect of the molecular model, a comparison between the corresponding velocity profiles was conducted. The comparison was done along the symmetry axis at the beginning (x=2 μm), the middle (x=5 μm) and the end (x=8 μm) of the mixing chamber for each model and the computed axial velocities are tabulated in Table 5. It can be seen that the associated reported relative velocity differences, also presented in Table 5, are very small up to approximately 3%. Thus, although the effect of the intermolecular model on the bulk velocities was small, its effect on the mixing process was significant. The authors in [15,16] arrived at the same conclusion by investigating different inlet bulk velocities. These findings, along with the conclusion by Koura and Matsumoto that the VSS should be preferred in gas mixture flows as a more reliable model [25], explains the reasoning behind considering the VSS model in the basic case flow scenario.

Finally, the effect of the molecular mass ratio of the two components of the mixture is considered. In Table 6, the mixing lengths $l_{\mathrm{mix}}^{a}$ are provided for the following combinations: CO-N_2_, Ne-Ar, He-Ne, He-Ar, and He-Xe. The corresponding molecular mass and diameter ratios of the light over the heavy species are depicted in the second and third columns respectively. In addition, the range of the $RDD^{a}$ in the region downstream of the corresponding mixing length is tabulated in order to demonstrate the mixture homogeneity, with the minimum values referring to the mixing length. It is seen that the mixing length of each component depends weakly on the molecular mass, with the mixing length of the light and heavy species varying by no more than 10%. Also, no conclusive comment can be made concerning the behavior of the mixing length with regard to the molecular mass, since, as the molecular mass ratio is decreased the mixing lengths of the species may either increase or decrease. It is also seen however, that the range of variation of $RDD^{2}$ is always larger than the corresponding $RDD^{1}$, except of course for the CO-N_2_ mixture, where the two molecular masses are the same. The values of $RDD^{1}$ and $RDD^{2}$ ranged from 0.5% to 1.0% and 0.5% to 2.4%, respectively, with the largest inhomogeneity referring to Xe. Actually, in the specific flow scenarios, if the threshold value is set to ε=0.5%, the mixture will never be considered fully mixed, because of the inhomogeneity of the heavy species of the mixtures, and the best possible mixing is the one demonstrated in Table 6. It is evident that the heavy species of the mixture are less uniformly distributed compared to the light species. In general, it can be stated that, independent of the mixture composition, the mixing lengths were approximately the same but with different homogeneity.

## 5. Micro-Mixer with Splitter

The analysis presented above provides a detailed view of the mixing process including the downstream composition of the mixture, based on all input parameters and conditions. However, having a solid knowledge of the output molar fraction of the mixture and furthermore controlling the output mixture composition, before the simulations are performed and concluded, is not a trivial task, even for a micro-mixer of specific geometry. This is due to the large number and range of the input parameters, which is typical in binary gas mixture simulations in the transition regime, making a complete parametrization study computationally very expensive. Obviously, controlling the mixing output is of major importance in technological applications. 

Arguing that the output composition of the mixture may be somehow correlated to the ratios of the input pressures or number fluxes of the species is, in general, erroneous. Such an argument is valid only if the components have equal molecular masses (e.g., CO-N_2_). Otherwise, due to the different molecular masses of the two species, this approach cannot be applied in the transition regime. This is clearly demonstrated in the following numerical experiment. Consider the mixing of He-Xe in the micro-mixer of Figure 1 with $P_{\mathrm{in}}^{\mathrm{He}}=0.2$ atm, ${\dot{N}}_{\mathrm{in}}^{\mathrm{He}}=623$ particles per cell area per time step, $T_{0}=T_{w}=300$ K, α=1 and the VSS model. Two different inlet conditions are considered: 

(a) the inlet pressure of the two species is the same, i.e., $P_{\mathrm{in}}^{\mathrm{He}}=P_{\mathrm{in}}^{\mathrm{Xe}}$, resulting in an inlet flux ratio equal to ${\dot{N}}_{\mathrm{in}}^{\mathrm{He}}/{\dot{N}}_{\mathrm{in}}^{\mathrm{Xe}}=6$;

(b) the inlet number fluxes of the two species are the same, i.e., ${\dot{N}}_{\mathrm{in}}^{\mathrm{He}}={\dot{N}}_{\mathrm{in}}^{\mathrm{Xe}}$, resulting in a pressure ratio equal to $P_{\mathrm{in}}^{\mathrm{He}}/P_{\mathrm{in}}^{\mathrm{Xe}}=0.175$.

**Figure 6:**
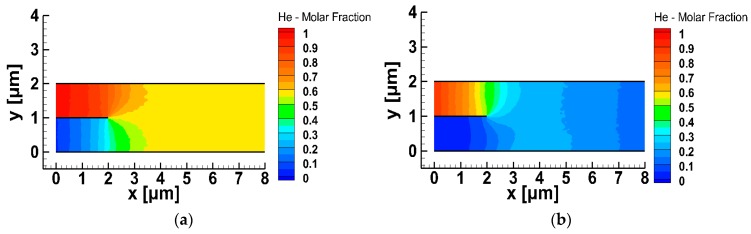
Molar fraction of He in the micro-mixer imposing: (**a**) equal inlet pressures of He and Xe, and (**b**) equal inlet fluxes of He and Xe.

The corresponding mixing results are presented in Figure 6a,b respectively, where the contours of the molar fraction of He in the micro-mixer are plotted. The downstream molar fractions of He, where the mixing criterion of RDD<0.5% has been fulfilled, are also provided and they are $C^{\mathrm{He}}=57.3\%$ and $C^{\mathrm{He}}=19.6\%$ in the cases of equal inlet pressures and fluxes, respectively. Then, the ratios of the molar fractions $C^{\mathrm{He}}/C^{\mathrm{Xe}}$ are found to be 1.35 in the case of equal inlet pressures (Figure 6a) and 0.243 in the case of equal inlet fluxes (Figure 6b). Both molar fraction ratios are completely different compared to the corresponding inlet flux and pressure ratios, which are equal to 6 and 0.175 respectively.

Based on all the above, a rather simple approach was proposed to control the output mixture composition, by modifying the device slightly (hardware part) and implementing a proposed algorithm (software part). As shown in Figure 2, a plate parallel to the others was positioned in the mixing chamber with its origin at some point (x,y)=(d+w,h), where $d<w<d+l_{\mathrm{mix}}$ and 0<h<2H. Since the flow is split into two mainstreams this plate was named the “splitter”. The mixture composition above and below the splitter differed from each other, as well as from the final fully mixed one without the splitter. Therefore, locating the splitter in various positions in the mixing chamber would result in different mixture compositions. It is noted that the splitter was considered without thickness and therefore, the proposed design, as depicted in Figure 2 and used in the simulations, serves mainly as a proof of concept. 

The next step was the cartography of the mixture molar fraction in the mixing chamber with the splitter in various positions, and then, the identification of the proper position of the splitter in order to provide a prescribed downstream mixture composition in a computationally efficient manner.

The proposed algorithm was demonstrated by considering mixing He and Xe in the flow configuration shown in Figure 2, positioning the splitter in 25 different locations. More specifically, the origin of the splitter was positioned at all the combinations of the following x,y coordinates:

x=3.50, 4.50, 4.75, 5.0, 5.25; y=0.2, 0.5, 1.0, 1.5, 1.8.

All other parameters remained the same as in the base case scenario. All 25 flow cases were simulated and the corresponding downstream molar fractions of He ($C^{\mathrm{He}}$) are tabulated, on a percentage basis, in Table 7 for the mixture above the splitter (upper outlet), and in Table 8 for the mixture below the splitter (lower outlet). Analyzing the tabulated results, it is seen that, depending on the splitter location, $C^{\mathrm{He}}$ varies from 27–52% and from 54–85% in the lower and upper streams, respectively. This is easily justified, since He is entering the micro-mixer from above and for the same reason, as y is increased, i.e., as the splitter is located closer to the upper plate, $C^{\mathrm{He}}$ is also increased. Also, as x is increased, $C^{\mathrm{He}}$ is increased, while the corresponding upper and lower values of $C^{\mathrm{He}}$ are closer to each other. Both observations are explained by the fact that as the splitter is located further downstream, the homogeneity of the mixture just before the splitter is increased. More importantly, it becomes clear that based on this concept, mixtures with a wide range of compositions may be produced.

**Figure 7:**
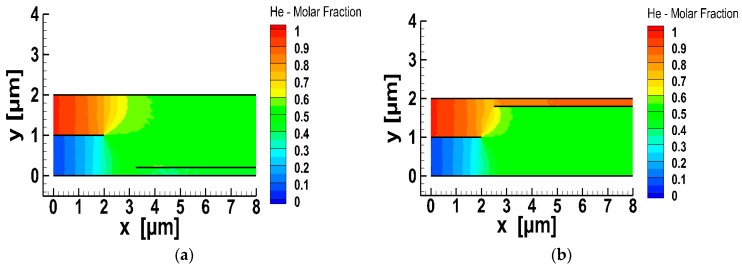
Gas mixing of He-Xe in the micro-mixer with splitter: Molar fraction contours of He with the splitter positioned at points (**a**) (3.25, 0.2) and (**b**) (2.5, 1.8).

Furthermore, in order to have a detailed view of the molar fraction filed in the micro-mixer with the splitter, in Figure 7 the contours of $C^{\mathrm{He}}$ are plotted for two indicative (out of the 25) flow cases, with the splitter origin located at (3.25, 0.2) and (2.5, 1.8). In both cases, the inlet, premixing and the two mixing areas resulting in mixtures of the same components but different molar fractions are clearly defined. When the splitter was positioned closer to the lower plate the mole fractions of the He-Xe mixture at the upper and lower outlets were 52.7% and 40.7% respectively, while when the splitter was positioned closer to the upper plate the corresponding values were 84.9% and 50%, respectively. When the splitter was positioned closer to the lower plate the mole fractions of the He-Xe mixture at the upper and lower outlets were 52.7% and 40.7%, respectively, while when the splitter was positioned closer to the upper plate, the corresponding values were 84.9% and 50%, respectively. These results were qualitatively expected since He is entering from the upper inlet. It is clearly seen that He-Xe mixtures in a wide range of mole fractions may be deduced. 

Next, the objective was to develop a simple computational tool capable of providing the correct position of the splitter once the desired outlet mixture composition was specified, without performing the whole simulation each time, which requires computing resources and is time consuming. Based on the data in Table 7 and Table 8, a least square approach in the x and y coordinates was implemented, to derive two third order polynomials, one for above and one for below the splitter, to approximate the molar fraction in the corresponding streams. The general form of the two polynomials is:

(6) $$C^{\mathrm{He}}(x,y)=a+bx+cy+dx^{2}+ey^{2}+fx^{3}+gy^{3}+hxy+ix^{2}y+jxy^{2}$$

where the coefficients of the two polynomials are given in Table 9. The relative error between the molar fractions provided by Equation (6) and the corresponding ones in Table 7 and Table 8 was less than ±3%, while the Euclidean norm of the error vector was approximately 2.8. The accuracy is considered as very good, and certainly much better than that obtained by other tested approximation formulas. 

**Figure 8:**
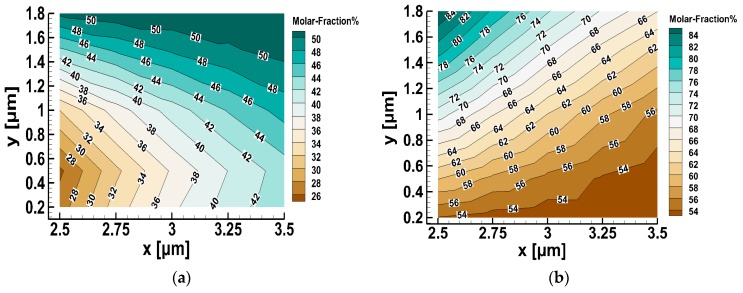
Molar fraction contours of He in the lower (**a**) and upper (**b**) outlets based on positioning the splitter in 25 different positions.

The contours of the molar fractions provided by the third order polynomials are provided for the lower and upper outlets in Figure 8a,b respectively, helping visualize the cartography of the molar fraction maps. The contours have been plotted in the same two dimensional space with the results in Table 7 and Table 8, i.e., for 2.5≤x≤3.5 and 0.2≤y≤1.8. The molar fraction step between the contours is small enough to allow an accurate estimation. 

**Figure 9:**
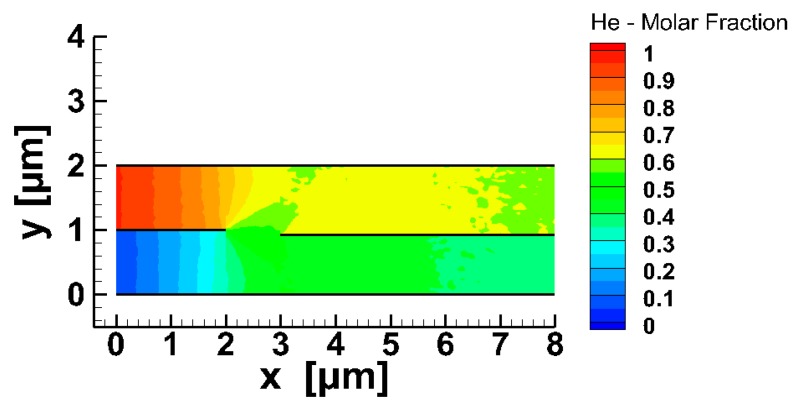
Molar fraction contours of He in the micro-mixer with the splitter positioned at (3, 0.93).

Once the desired mixture composition was prescribed, then based on Equation (3) for the molar fraction and the associated contours of Figure 8, it was possible to estimate the proper location of the splitter in order to obtain the specified composition, as follows:

Assume that the specified downstream molar fraction of He is $C^{\mathrm{He}}=40\%$. Observing the contours in Figure 8, it is easily seen that the specified composition may be obtained only in the lower outlet stream and for (x,y) coordinates deducing a molar fraction at contour line 40%. In order to ensure the validity of the approach, one of the two coordinates was fixed, e.g., x=3 μm and Equation (7) for the lower outlet was solved by an iterative method to find y=0.93. This result was next validated by running the whole simulation for the flow field in the micro-mixer with the origin of the splitter located at (3, 0.93). The computed $C^{\mathrm{He}}$ field is shown in Figure 9. At the lower outlet, the downstream molar fraction of He was computed to be 40.7%, which is only slightly different than the specified one. The introduced discrepancy is due to the small error introduced in the regression model.

Obviously, the approximate expression (6) is valid only for He-Xe mixing but the proposed methodology can be also applied in other gas mixtures, depending upon the technological application.

## 6. Summary and Concluding Remarks

The mixing process of two pressure driven steady-state rarefied gas streams flowing between two parallel plates was investigated. The micro-mixer assembly was simple and consisted of the inlet and mixing zones. Upon entering the mixing zone, gas mixing progressed very rapidly and then it slowed down, reaching in an asymptotic manner, at some distance from the inlet zone, the optimum mixture homogeneity. This distance was defined as the mixing length. Then, further downstream, the homogeneity of the mixture slightly deteriorated up to the end part of the channel, where the homogeneity decreased more rapidly, due to gas separation and end effects. The homogeneity of the mixture was evaluated based on the relative difference between the species number density at each cell of a channel cross section and the corresponding average number density of the cross section. In general, gas mixing is upgraded as the mixing length is decreased and the relative density difference is decreased, otherwise the mixture homogeneity is increased.

The mixing length, with the associated relative density difference, was computed for different combinations of gases, in terms of the parameters affecting the mixing process, in a micro-mixer assembly having length and height equal to 8 μm and 2 μm, respectively. The mixing length, in most flow scenarios, varied between 2 μm and 4 μm. It has been shown that increasing the channel length does not affect the mixing length and the associated relative density difference, i.e., the grade of the mixture homogeneity. Varying the wall temperature also has a minor effect on the gas mixing. The type of gas surface interaction is the most important parameter affecting gas mixing and more specifically, as the reflection becomes more specular, the mixing length significantly increases. In cases with high specular reflection, the mixing length may be doubled or even tripled. The intermolecular collision models of HS, VHS and VSS were implemented and it was found that the computed mixing length of the HS and VHS models were about 20% and 10% higher than for VSS, while the corresponding relative density differences were negligible. It is noted that in gas mixture modeling the VSS model is considered the most reliable one. Concerning the mixture composition, it was deduced that the ratio of the molecular masses of the two components ranged, in the present study, from 1 down to 0.03, and had a minor effect on the mixing length and a more important one on the relative density difference. More specifically, the mixture became less homogenous as the molecular mass ratio reduced, i.e., the molecular masses of the two species are quite different.

Next, a rather simple approach has been proposed to control the output mixture composition. A plate, parallel to the others, the so-called splitter, was added in the mixing zone of the micro-mixer assembly, separating the downstream flow into two outlet mainstreams. It has been shown that locating the splitter in various positions in the mixing zone will result in different downstream mixture compositions. Intensive simulations were performed for 25 different positions of the splitter and the molar fractions of the produced binary mixtures above and below the splitter have been deduced. Based on these data, simple approximate expressions have been derived capable of providing, once the desired outlet mixture composition is specified, the correct position of the splitter, without performing time consuming simulations. The analysis has been performed and validated for He-Xe flow but can also be easily applied to other gas mixtures.

It is hoped that the mixing analysis performed and the proposed approach for controlling gas mixing in order to obtain binary gas mixtures with specific compositions, will support corresponding experimental work, as well as the design and optimization of gas mixing micro devices.

## Figures and Tables

**Table 1:** Direct Simulation Monte Carlo (DSMC) properties.

Property	Value
Collision scheme	No Time Counter (NTC)
Molecular model	VSS (monoatomic)
Time averaging	20 kinetic steps
Total samples	500 (1 sample = 20 kin. steps)
Ensemble averaging	50 (simulations)

**Table 2:** Effect of the wall temperature on the mixing length for CO-N_2_ flow.

$T_{w}$ (K)	$l_{\mathrm{mix}}$ (μm)	$C^{1}$	$RDD^{1}$	$RDD^{2}$	〈RDD〉
300	2.8	50.1%	0.59%	0.55%	0.76%
450	2.7	49.9%	0.75%	0.59%	0.98%
600	2.5	50.1%	0.64%	0.75%	1.0%

**Table 3:** Effect of the wall temperature on the mixing length for He-Xe flow.

$T_{w}$ (K)	$l_{\mathrm{mix}}$ (μm)	$C^{1}$	$RDD^{1}$	$RDD^{2}$	〈RDD〉
300	2.2	57.7%	0.83%	1.16%	0.93%
450	2.3	57.9%	0.87%	0.95%	0.98%
600	2.2	57.6%	0.85%	1.02%	1.04%

**Table 4:** Effect of the molecular model on the mixing length for CO-N_2_ flow.

Molecular Model	$l_{\mathrm{mix}}$ (μm)	$C^{1}$	$RDD^{1}$	$RDD^{2}$	Relative Difference of the HS and VHS with Respect to the VSS
HS	3.3	49.8%	0.60%	0.55%	17.4%
VHS	3.15	50.1%	0.59%	0.53%	12.4%
VSS	2.8	50.1%	0.59%	0.55%	-

**Table 5:** Effect of the molecular model on the mixture velocities at several locations along the symmetry axis for CO-N_2_ flow.

x (μm)	Velocity (× 422.08 m/s)			Relative Velocity Difference		
	VSS	VHS	HS	$\frac{\left|\mathrm{VSS}-\mathrm{VHS}\right|}{\mathrm{VSS}}$	$\frac{\left|\mathrm{VSS}-\mathrm{HS}\right|}{\mathrm{VSS}}$	$\frac{\left|\mathrm{VHS}-\mathrm{HS}\right|}{\mathrm{VHS}}$
2	0.1837	0.1885	0.1858	2.61%	1.14%	1.43%
5	0.3133	0.3232	0.3172	3.16%	1.24%	1.88%
8	0.7692	0.7785	0.7682	1.21%	0.13%	1.32%

**Table 6:** Effect of the molecular masses of the mixture components on the mixing length.

Species	$m_{1}/m_{2}$	$D_{1}/D_{2}$	$RDD^{1}$ Range Downstream of the Mixing Length	$l_{\mathrm{mix}}^{1}$ (μm)	$RDD^{2}$ Range Downstream of the Mixing Length	$l_{\mathrm{mix}}^{2}$ (μm)
CO-N_2_	1.000	1.002	0.5–0.7%	2.83	0.5–0.7%	2.83
Ne-Ar	0.506	0.659	0.7–1.1%	2.23	1.0%	2.16
He-Ne	0.200	0.845	0.7–1.0%	2.30	1.0–1.5%	2.50
He-Ar	0.100	0.559	0.5–1.1%	2.30	1.1–1.6%	2.56
He-Xe	0.030	0.404	0.6–1.0%	2.23	1.4–2.4%	2.16

**Table 7:** Downstream molar fraction (%) of He at the lower outlet for 25 different positions of the splitter.

	*x* (μm)	2.5	2.75	3	3.25	3.5
*y* (μm)						
0.2		27.84	32.92	37.16	40.68	43.4
0.5		24.81	31.18	36.31	40.45	43.47
1		31.11	37.35	41.6	44.49	46.55
1.5		44.87	46.75	48.13	49.12	49.79
1.8		50.05	50.89	51.18	51.37	51.47

**Table 8:** Downstream molar fraction (%) of He at the upper outlet for 25 different positions of the splitter.

	*x* (μm)	2.5	2.75	3	3.25	3.5
*y* (μm)						
0.2		54.22	53.62	53.13	52.76	52.47
0.5		58.82	56.81	55.3	54.13	53.28
1		72.15	65.49	61.64	58.26	56.01
1.5		81.66	75.11	69.33	64.62	61
1.8		84.92	79.85	74.73	70.08	66.18

**Table 9:** Coefficients of the third order polynomials for the upper and lower outlet.

Coefficient	Lower Outlet	Upper Outlet
*a*	−1.4699150657035133 × 10^2^	6.5921177492441657 × 10^1^
*b*	1.2957775301457653 × 10^2^	−1.5812706830864261 × 10^1^
*c*	−2.0134328955701239 × 10^1^	1.1039559381541720 × 10^2^
*d*	−2.8591563402844741 × 10^1^	4.2562568218902328 × 10^0^
*e*	6.1478013059979929 × 10^1^	−1.4992529652493928 × 10^1^
*f*	2.0906666666617326 × 10^0^	−1.8133333333999069 × 10^−1^
*g*	−8.6358974358975775 × 10^0^	−2.5153846153845123 × 10^0^
*h*	−1.1499560838143527 × 10^1^	−4.3247339251155680 × 10^1^
*i*	3.5492776886033468 × 10^0^	2.4340288924554212 × 10^0^
*j*	−1.0025917815277760 × 10^1^	8.3286615998969680 × 10^0^

**Figure A1:**
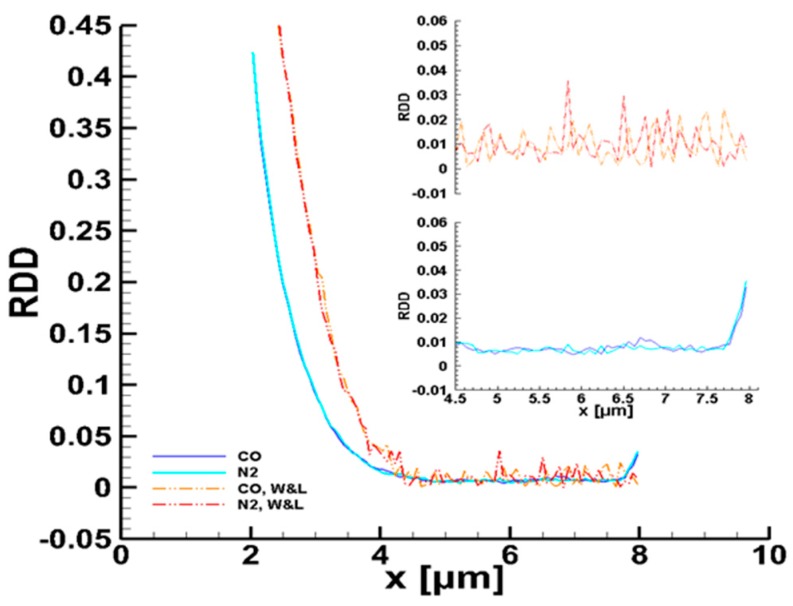
Distribution of relative density difference of CO and N_2_ calculated with the present definition and Wang and Li’s definition in [15]; the results are zoomed for a specific channel length at the upper right.

## Appendix A

The RDD expression introduced by Wang and Li [15] is given by:

(A1) $$\xi_{j}^{a}=\frac{\left|n_{\mathrm{up},j}^{a}-n_{\mathrm{low},j}^{a}\right|}{\max\left(n_{\mathrm{up},j}^{a},n_{\mathrm{low},j}^{a}\right)}$$

where, $\xi_{a}^{j}$ is the relative density difference $RDD^{a}$, of species a=1,2 at column j, while $n_{\mathrm{up},j}^{a}$ and $n_{\mathrm{low},j}^{a}$ correspond to the number densities of the species in the same column at the two cells adjacent to the upper and lower walls, respectively. The RDD definition, implemented in the present work (see Equation (1)), instead of only the two cells adjacent to the upper and lower walls, considers all column cells. A comparison between the two definitions in terms of the corresponding distribution of the RDD along the channel was performed.

Consider the steady-state mixing flow of CO-N_2_ in the micromixer of Figure 1, with $P_{\mathrm{in}}^{1}=P_{\mathrm{in}}^{2}=0.2$ atm, $T_{0}=T_{w}=300$ K and purely diffuse reflection at the walls. The computed distributions of the relative density differences by the present expressions and the one in [15] along the channel are plotted in Figure A1. Simulations were performed by setting $RDD^{a}<\varepsilon$, with ε=0.5%. The $RDD^{a}$ distributions of both species, a=1,2, obtained by the two definitions were qualitatively similar and more important the computed mixing length was the same and equal to $l_{\mathrm{mix}}=2.8$ μm. However, the introduced DSMC statistical noise, which is a very significant quantity in probabilistic computations, was much higher in the simulations via the expression in [15]. This is more clearly shown in the detail of Figure A1, where the evolution of *RDD* from a length near the mixing point until the end of the channel, as calculated in [15] and by Equation (1) respectively, are plotted.

Furthermore, the fact that the variation of $RDD^{1}$ is identical to $RDD^{2}$ is due to both having the same molecular masses of CO and N_2_, while the sudden increase of both $RDD^{a}$ at the channel end is due to gas separation and end effects, an effect that is only captured by the proposed method. Therefore, in the present work, the RDD is computed according to Equation (1). These issues are further discussed in Section 4.
